# Breakdown of Diabetic Foot Ulcer Care during the First Year of the Pandemic in Poland: A Retrospective National Cohort Study

**DOI:** 10.3390/ijerph19073827

**Published:** 2022-03-23

**Authors:** Marcin Kleibert, Beata Mrozikiewicz-Rakowska, Patrycja Małgorzata Bąk, Daniel Bałut, Jakub Zieliński, Leszek Czupryniak

**Affiliations:** 1 Department of Diabetology and Internal Diseases, Medical University of Warsaw, 02-097 Warsaw, Poland; patrycjambak@gmail.com (P.M.B.); daniel.balut@interia.eu (D.B.); leszek.czupryniak@wum.edu.pl (L.C.); 2 Interdisciplinary Centre for Mathematical and Computational Modeling, University of Warsaw, 02-106 Warsaw, Poland; jziel@op.pl

**Keywords:** COVID-19, diabetic foot ulcer, SARS-CoV-2, epidemiology, mortality, pandemic, amputation, death, lockdown, healthcare system, public health

## Abstract

The COVID-19 pandemic revealed a breakdown of the system of DFU patient care. This retrospective national cohort study analyses the epidemiological status of DFU patients in relation to urgent and elective hospitalizations, amputation rates, and deaths in Poland from 2017 to 2019, and during 2020 when the COVID-19 pandemic began. The data were obtained from national medical records gathered by the National Health Fund (NHF). Discharge diagnoses were categorized according to ICD-10 and ICD-9 codes. Analysis of the data showed a statistically significant decrease in elective hospital admissions (from 29.6% to 26.3%, *p* = 0.001). There was a decrease in the percentage of hospitalizations related to limb-salvage procedures (from 79.4% to 71.3%, *p* = 0.001). The opposite tendency was observed among urgent hospital admissions (from 67.0% to 73.2%, *p* = 0.01), which was related to a significant increase in the number of minor amputations (from 3146 to 4269, *p* = 0.017). This rise was in parallel with the increase in the percentage of patients who died during hospitalization due to DFU (from 3.9% to 4.8%, *p* = 0.03). The number of deaths has not changed significantly (from 590.7 to 668.0, *p* = 0.26). The results of the conducted analyses confirm the negative tendencies in the medical care of patients with DFU during the first year of the pandemic in Poland. Changes in therapy schemes and stronger patient support following this period are necessary to avoid further complications in patients with DFU.

## 1. Introduction

The COVID-19 pandemic has had an enormous impact on patients with DM [1]. Within the first months after the first confirmed case of SARS-CoV-2 in China, diabetic and obese patients formed one of the largest groups among those hospitalized [2,3]. After the first reports of an unknown case of pneumonia in December 2019, a pandemic and health alert were announced. This resulted in the limitation of accessibility to care in many countries due to the fear of infection and spread of the disease during the whole of 2020 [4]. In view of these facts, the impact of the COVID-19 pandemic on the healthcare system should be analyzed from the beginning of 2020.

Additionally, compliance among diabetic patients with medication was significantly reduced during and after lockdown due to limited contact with a doctor. Moreover, patients were not familiarized with healthy lifestyle habits [5]. Surprisingly, some studies showed that glycemic control has not changed and even improved during lockdown [6,7,8,9]. It was also confirmed that patients with diabetes had a higher risk of developing COVID-19 and diabetes-related late complications during the pandemic [10]. Moreover, the presence of a COVID-19 infection may induce thrombotic complications within the course of diabetes [11]. Due to this, patients with diabetes became one of the highest risk groups for fatal outcomes related to COVID-19 infection [5,12].

Among diabetic complications, diabetic foot ulcer (DFU) appears to be one of the most traumatizing. Additionally, DFU is one of the most challenging problems among patients with diabetes mellitus for public health. This complication is the most common cause of hospital admissions in Western countries related to DM [13]. The breakdown of healthcare systems around the world during the COVID-19 pandemic especially affected this group of patients [14]. The first data about the increased number of amputations due to DFU during the pandemic came from Italy and the United States [15,16]. These were related to the reduction in the capacity of hospitals due to the increased number of patients infected with SARS-CoV-2 who needed specialist care [17]. The shift from traditional to online visits in outpatient clinics was difficult for the elderly who constitute the biggest group of patients with DFU [18]. Additionally, this shift was challenging for patients with DFU because these patients require face-to-face interaction for wound debridement and dressing changes, which were difficult during the pandemic [19]. The epidemic safety regulations, such as restricted outside activity, resulted in limited exercise, irregular diet, and poor self-management of patients [5]. All these changes may lead to worse outcomes related to the worsening of the wound healing process among patients with DFU compared with the pre-COVID era [14]. However, it appears that lockdown and the restrictions of outdoor activity reduced the risk of trauma and DFU occurrence which may have resulted in a lower number of newly diagnosed patients with DFU. As mentioned above, some studies showed, for example, that glycemic control improved during the lockdown, so it is important to verify whether the impact of the pandemic was negative or positive for patients with DFU [5,6,7,8,9]. We would like to present the first data from the national registry gathered from the Polish population comparing the number of hospitalizations, amputations, and death rate among patients with DFU in 2020 in a country with a relatively low healthcare expenditure in comparison with Western Europe and the United States. These data can help to check the real impact of the pandemic on patients with DFU. In addition, potential solutions for reducing mortality in this group of patients are presented.

## 2. Materials and Methods

### 2.1. Study Design

The data of elective and urgent hospitalizations related to procedures coded as 86.221–223, 84.119, and 84.129, along with the number of amputations, limb-saving procedures, and deceased patients due to DFU were obtained from the National Health Fund (NHF), which is the institution responsible for the collection of medical records of all patients discharged from public hospitals and other health service institutions in Poland. All the data are compiled at the national level. The Polish Wound Management Association has recommended that the mode of admission should be established according to IDSA/IWGDF [20]. Most patients with the fourth stage of DFU infection were admitted in urgent mode during the pre-pandemic period, whereas from 2020 this was extended to also cover the third stage. Additionally, patients were qualified for urgent (third and fourth stages) or elective (second stage in case of the lack of progress in healing or vascular surgery intervention) admission based on a telephone consultation in most cases in 2020. Discharge diagnoses were recorded by physicians during the hospital stay and coded according to the 10th Edition of the International Classification of Diseases (ICD-10). Patients with diabetes were identified as E10–E14 based on the ICD-10 classification and with DFU as L97. We omitted cases of patients with DFU coded other than L97 to reduce errors related to wrong classification. The procedures were analyzed according to the 9th Edition of the International Classification of Diseases (ICD-9). The limb-salvage procedures among patients with DFU were defined as 86.221–86.223 (any intervention in the foot area not resulting in an amputation, such as removal of devitalized tissue or topical treatment), which can be performed also among patients before amputation. Minor amputations were defined as amputations below the ankle and were coded as 84.119 and 84.129. Major amputation was defined as amputations below or above the knee and was coded as 84.151, 84.171, 84.172, and 84.174. Diabetic amputations were defined as those performed in patients with diabetes, but not earlier than 30 days before its diagnosis. All procedures were analyzed among the combined group of urgent and elective admissions.

This nationwide cohort study analyzed data collected in the period between 1 January 2017, and 31 December 2020. Data obtained between 1 January 2017, and 31 December 2019, were combined and averaged to minimize the error related to annual fluctuations. This was defined as COVID = 0 and this designation is presented in the charts and tables. Next, a comparison with the period between 1 January and 31 December 2020, marked as COVID = 1, was performed. We analyzed the whole of 2020 because, to the best of our knowledge, the problems with the availability of healthcare and DFU-care began in January in Poland. However, the first confirmed case of SARS-CoV-2 in Poland was announced on 4 March 2020.

### 2.2. Statistical Analysis

Data were analyzed using Statistica 13 (TIBCO Software Inc., Palo Alto, CA, USA). Differences between multiple groups (2017–2019 vs. 2020) were evaluated using a One-way and Two-way ANOVA test. Statistical significance was set at a *p*-value < 0.05.

## 3. Results

### 3.1. Patient Characteristics

The total number and characteristics of patients did not differ significantly between the 2017 to 2019 (combined and averaged data) and 2020 (Table 1). Most patients were men (65.3% in the pre-pandemic period and 67.1% in 2020). The largest group of hospitalized patients was between 61 and 80 years old (52.7% in the pre-pandemic period and 55.3% in 2020).

### 3.2. Number of Hospitalizations

**Figure 1:**
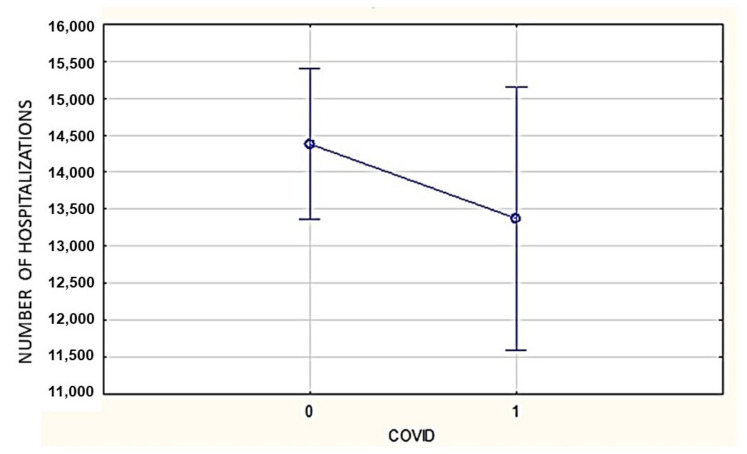
The number of DFU-related hospitalizations in Poland (2017–2019 (combined and averaged data), COVID = 0 vs. 2020, COVID = 1; 14,382.7 vs. 13,375, *p* = 0.17).

The number of DFU-related hospitalizations did not change significantly in 2020 in comparison with 2017–2019 (combined and averaged) (13,375 vs. 14,382.7; *p* = 0.17) (Figure 1).

### 3.3. Mode of Admission of Patients 

The mode of admission has changed significantly during the pandemic. A lower percentage of patients was admitted to hospital as elective cases in 2020 in comparison with the pre-pandemic period (26.3% vs. 29.6%, *p* = 0.001) (Table 2). The opposite trend was observed for urgent admissions (73.2% vs. 67%; *p* = 0.01) (Table 2). These differences were statistically significant.

### 3.4. DFU-Related Amputations

The number of all amputations has not significantly changed in the analyzed period in Poland (5292 in 2017–2019 (combined and averaged data) vs. 5869 in 2020, *p* = 0.13) (Table 3). However, we observed an increasing tendency. The number of minor amputations (below the ankle) increased by 35.7% in 2020 (3146 in 2017–2019 (combined and averaged data) vs. 4269 in 2020, *p* = 0.017) (Table 3). Simultaneously, the number of major amputations (above the ankle) decreased during the pandemic, but it is at the limit of statistical significance (2146 in 2017–2019 (combined and averaged data) vs. 1600 in 2020, *p* = 0.05) (Table 3).

### 3.5. Limb-Salvage Procedures

The limb-salvage procedure was performed in a lower percentage of cases in 2020 compared with 2017–2019 (71.3% vs. 79.4%; *p* = 0.001) (Table 3). The number of performed procedures did not change significantly (7488 vs. 8355.7, *p* = 0.15).

### 3.6. Mortality

The percentage of patients who died during DFU-related hospitalization increased by 34.4% in 2020 in comparison with the pre-pandemic period (4.84% vs. 3.9%; *p* = 0.03) (Table 4). The number of these patients did not change significantly, but an increasing tendency was observed (668.0 vs. 590.7, *p* = 0.26).

## 4. Discussion

The aim of the study was to assess the impact of the COVID-19 pandemic on mortality and the number of procedures (amputation and limb-salvage procedures) related to DFU in 2020 in Poland. We observed an increase in urgent hospitalization due to DFU with a simultaneous increase in minor amputations and a decrease in major amputations. Additionally, we noted a decline in elective admissions and limb-salvage procedures performed within the observational period. The ultimate consequence was an increase in the mortality rate, which confirms the necessity of urgent remodeling of care dedicated to patients with DFU in Poland.

The experiences of the first year of the COVID-19 pandemic revealed many shortcomings and an insufficiency of the healthcare system which made an enormous impact on the effectiveness of treatment of patients with chronic diseases such as DM [5,21,22]. The implications of this sudden change in hospital and ambulatory care systems probably had the biggest influence on the occurrence of complications of these diseases, e.g., DFU [23]. It appears that limitations in hospital admissions might be compensated by the relocation of patients to outpatient clinics. Surprisingly, the opposite occurred, namely, in one of the DFU outpatient clinics in Slovakia, a decline of 17.8% in the number of visits was observed [24]. A few countries noted a significant decline in both hospital and ambulatory care, even related to acute conditions such as stroke and myocardial infarction [25,26,27].

This situation also affected countries with well-organized healthcare systems. The COVID-19 pandemic brought unexpected changes in previously very-well-organized systems, which influenced management, access to training, and education. Even countries such as Italy and Germany which, according to the reports of the Organization for Economic Cooperation and Development, were listed as having exemplary ambulatory care for patients with DFU resulting in low amputation rates, experienced a breakdown in care [15,28,29].

Schlager et al. reported that in Germany the pandemic has not had a significant negative impact on the quality of ambulatory care dedicated to patients with DFU (*n* = 63) [30]. Unfortunately, the authors only assessed the soft endpoints, such as the quality of life and the frequency of changing wound dressing. Fourteen percent of patients experienced delays or cancellations of diagnostic workup or hospitalization during the lockdown. Alternative solutions such as telemedicine were not often used by patients and physicians to ensure continuity of care in this study [30]. In view of these facts, a redefinition of DFU management service during the COVID-19 pandemic is needed. The physicians have to change their mode of healthcare delivery and patients have to face the challenge of DFU self-monitoring to improve future outcomes [30].

A survey conducted around the world between December 2020 and March 2021 among doctors (*n* = 1478) specializing in treating DFU patients proves the breakdown of the care system for those patients [31]. Moreover, diabetes nurses (*n* = 1829) observed the same negative changes in the quality of care among patients with complications of DM. They noticed an increase in the number of patients with anxiety and depression [32]. In an Italian survey, completed by 34 medical doctors and 12 nurses, 76.1% of the responders answered that the pandemic affected the management of wound dressings [33]. The same problems were observed in India, where 72.7% (*n* = 24) of the doctors recorded difficulties during inpatient consultations [34]. Changes were inevitable even in the United States. A recent analysis has shown that more than 40% of wound care practices were closed for part of 2020, which directly made accessibility to wound care even more difficult [35]. In several countries, a breakdown in ambulatory admissions was noted. The lockdown in South Africa serves as an example of this practice. There, almost all in-person ambulatory visits were stopped and patient care was delayed for months [36]. Poland had the same difficulties in the functioning of the healthcare system during the first waves of the COVID-19 pandemic [37]. Most patients with chronic illnesses usually make use of the public healthcare system in Poland. The Central Statistical Office reported 256.6 million medical and 26.5 million dental appointments in 2020, which indicates a 20% decline in comparison with 2019. More than 36% of the medical appointments were conducted virtually, with the use of telemedicine devices [38].

In the analyzed data from the presented study, a substantial decline in the percentage of elective admissions and an analogous increase in urgent admissions were noted (Table 2). The Polish government recommended reducing the number of elective admissions and non-urgent interventions (limb-salvage procedures), which might explain this tendency. Urgent admission of patients was possible throughout the whole of the pandemic. In one of the Italian tertiary care centers, an increased percentage of urgent admissions related to DFU was also observed in 2020 in comparison with 2019 (76% vs. 26%, *p* = 0.001) [15]. Mariet et al. reported a decline of 25.2% (*p* < 0.0001) in the total number of hospital admissions related to DFU during the pandemic in comparison with a similar period in 2019. Moreover, a decrease in hospitalizations due to lower limb amputations (11%, *p* < 0.0001), revascularizations (12%, *p* < 0.0001), and osteomyelitis (23%, *p* < 0.0001) was noted [23]. In our analysis, the total number of admissions has not changed significantly, which might be due to the worse organization of hospital care and a lower number of hospital beds dedicated to patients with DFU in the pre-pandemic period in Poland. Additionally, many patients were treated in outpatient clinics in the pre-pandemic period, which were closed in 2020. These patients should then be referred to hospital. However, we observed a simultaneous further reduction in the number of clinics and hospital beds dedicated to patients with DFU (they were used for treating the patients with COVID) which resulted in an unchanged number of hospitalizations, because these patients could not be admitted to hospital due to lack of places [39].

At the beginning of the COVID-19 pandemic, it appeared that lockdown and restrictions on outdoor activity would positively influence the healing process in patients with DFU, and thus reduce the number of amputations. This thesis was verified after a short time. The data documented by a trauma center in Ohio showed that there was a statistically significant drop in the percentage of patients being admitted without infection due to diabetic foot (18.3% in the pre-pandemic period vs. 7.5% during the pandemic, *p* = 0.04) [40]. Additionally, an increase in the prevalence of mild (35.4%) and severe (15%) infections in comparison with the pre-pandemic period (29.6% and 9.6%) was recorded. This vastly different outcome indicates that patients with DFU were left without sufficient wound care for too long. In this study, the severity of infection has not been assessed, but the decline in the quality of care can be confirmed by an increased percentage of patients who died during hospitalization and a higher number of minor amputations.

The described relationship cannot fully reflect the actual worldwide state of patients with DFU. In many countries, there are different care systems designed to deal with DFU, which may vary from region to region. These health care systems have various efficacies of treatment within a single country [41]. In addition, every country had to face various difficulties related to the COVID-19 pandemic which forced changes in the healthcare system.

In many countries, there are diabetic foot centers which function as a place where a patient can undergo minimally invasive surgical treatment without the need for hospitalization. The abovementioned center in Ohio is an example of such a solution [40]. In Poland, this kind of care has not been developed. In most cases, such invasive procedures cannot be performed in outpatient clinics within the scope of the state health insurance system. Even systems of negative pressure therapy, apart from hospitals, are not usually used in public outpatient clinics [39]. Due to this fact, during the pandemic, we observed an enormous decrease in the percentage of limb-salvage procedures among DFU patients in Poland with a simultaneous increase in the number of minor amputations. This was caused by a delay in diagnostic procedures and the treatment of patients at the early stages of the infection process and DFU development. As a consequence, a significant increase in urgent hospital admissions was observed. The decrease in limb-salvage procedures proves the breakdown of DFU patient care.

In Poland, we observed an increasing trend in the total number of amputations due to DFU compared with the pre-COVID era (5292 in 2017–2019 (combined and averaged data) vs. 5869 in 2020, *p* = 0.13). This change was not significant due to the opposite tendency in the number of major and minor amputations. The number of major amputations (above or below the knee) in Poland decreased by 546 (*p* = 0.05). Simultaneously, we noted an increase in minor amputations (below the ankle) (3146 in 2017–2019 vs. 4269 in 2020, *p* = 0.017), which means that these differences cancel each other out and the change in the total number of amputations has not yet reached the threshold of statistical significance. The non-significant decrease in major amputations can also be explained by earlier admission due to the fear of the consequences during the pandemic and telecare. Despite the fact that telecare showed a similar efficacy in some trials and meta-analysis, this data cannot be applied to Poland because only phone calls are widely used by medical staff [42,43]. Due to this fact, it was hard to assess the actual status of patients during virtual contact which was made with the use of phone calls. Medical staff in emergency rooms were alerted to the potential thrombotic complications of COVID-19, observed in DFU patients. This did not come as a surprise in our country as healthcare expenditure is very low. In 2020, it was PLN 121.5 billion, which constituted 5.2% of the GDP (gross domestic product) [44]. Similar increases were also observed in countries with well-developed care systems for DFU patients. In an Italian epidemiological study describing the situation of these patients, the authors reported that the number of amputations in 2020 was also higher in comparison with previous years (60% vs. 18%, *p* = 0.001) [15]. In the Netherlands, there was also a significant increase from 18% in 2019 to 42% in 2020 in the number of major amputations [45]. In Poland, we observed a non-significant decrease in the number of major amputations with a simultaneous non-significant increase in the number of all (major and minor) amputations, which can be related to a two times higher risk of amputation in the pre-pandemic period in comparison with western countries, such as Italy [29]. The same increasing tendency was documented in China and India [46,47]. Reports from the United States also showed a 10.8 times increase in the risk of any amputations in patients with this diabetes complication (*p* < 0.0001; 95%, CI:6.5–17.8) [40]. In our analysis, we observed a significantly higher percentage (35.8%) of minor amputations. Only Valabhji et al. noticed a drop of 7% and 21% in major and minor amputations during the pandemic [48]. We observed a similar tendency among major amputations in our analysis.

Therefore, there is an urgent need to intensify the ways to reach those patients and to raise awareness among doctors specializing in DFU treatment to help increase the chances of the survival of these patients. The recommendations of D-Foot International during the COVID-19 pandemic highlighted the problem of potentially mild infections which may initiate a cascade leading to amputation or even death [49]. Based on the guidelines of international associations, there is an urgent need for new triage pathways among patients with DFU. A great example is the method introduced in Italy by Meloni et al. who showed that it could reduce mortality related to delay at the beginning of DFU treatment. The authors created an algorithm dedicated to patients with DFU which made it possible to choose the most appropriate management (ambulatory care—Traditional or virtual—Or hospitalization). This triage pathway uses a grading score for the severity of the ulcers and the number of comorbidities of the patient and can be easily used in daily practice [50]. Another example is the STRIDE protocol designed by Schmidt et al., which makes it possible to maintain a low rate of minor amputation and DFU-related hospitalization among patients with DFU during lockdown in comparison with the pre-pandemic period (20% vs. 24%, *p* > 0.05) [51]. The third algorithm was designed by Kelahmetoglu et al. [52]. Rastogi et al. also showed that the immediate use of the virtual triage among patients with DFU can reduce the negative impact of the pandemic on the outcome for patients [53]. All proposed solutions highlight the need for proper risk stratification. Home care has to be more important in the newly proposed management of DFU. The role of telemedicine in this area is still underestimated, and we have to be aware that in a short time this has to change [54]. An internet-based algorithm, such as that developed by Liu et al., may help in the better allocation of patients with DFU and optimize medical resources [55].

All the evidence indicates that the healthcare system was not prepared for the challenges caused by SARS-CoV-2. Ambulatory care could not compensate for the lack of specialist care. As a consequence of the breakdown, Poland recorded a dramatic increase in the percentage of deaths among hospitalized patients related to DFU. The relatively low percentage of in-hospital mortality in comparison with Nigeria (21.4%) in the pre-pandemic period might be connected with the better organization of DFU care [56]. There is no other data which compares in-hospital mortality related to DFU during the pandemic with the pre-pandemic period. In one study, the authors revealed that the mortality rate among non-hospitalized patients has not changed during the pandemic (3.8% in 2019 vs. 4.3% in 2020, *p* = 0.532) [53]. However, ambulatory patients are in a better condition than hospitalized, so this result cannot be compared with our results. Additionally, Rastogi et al. used a virtual triage to divide patients with newly diagnosed DFU according to their condition which has an impact on patient outcomes (no differences in the mortality rate and amputation risk) [53]. In our study, we also observed a non-significant increase in the number of amputations (from 5292 in 2017–2019 to 5969 in 2020, *p* = 0.13). The statistically non-significant increase can be related to the lower number of hospitalizations during the first year of the pandemic (14,382.7 vs. 13,375, *p* = 0.17).

We recognize that our study has several limitations, such as its retrospective nature, and errors which can be connected with the incorrect classification of patients during hospitalization. This error is associated with the fact that some specialists might incorrectly classify the procedure or intervention during hospitalization. However, this should not affect the study results significantly, because according to the NHF a similar number of incorrectly classified patients is reported each year, so its influence on the final result should cancel each other out. Moreover, the lack of more precise information about patient status, such as the presence of Charcot osteoarthropathy or sepsis during hospitalization may be recognized as another limitation. The fact that we analyze the whole of 2020 and not a specific period can be considered to be a limitation and should be taken into consideration during the interpretation of the results which we believe are a consequence of the COVID-19 pandemic. However, we consider that the COVID pandemic started to influence the healthcare system much earlier than before the first confirmed case. Using combined data from the whole of 2020, not only from 4 March, 2020, might have an impact on the results but, as mentioned, the reorganization began with the first days of 2020 in Poland, which was defined as the replacement of departments with dedicated ones for patients with COVID, a reduction in elective admissions, and a change of the form of outpatient consultations from traditional to virtual (mostly phone calls).

## 5. Conclusions

There is an urgent need for the reorganization of the care system for patients with DFU in Poland. It may help to avoid an unnecessary number of especially minor amputations and decrease the percentage of dying patients. Changes in the organization of care for patients with DFU are urgently needed. The proper triage of patients according to recently published guidelines is important to select patients who require specialist consultation and initiation of treatment in a shorter time when there are fewer appointments available.

## Figures and Tables

**Table 1:** Characteristics of patients enrolled in the analysis in 2017–2019 and 2020.

Variables		COVID = 0	COVID = 1	*p*
		*n* (%)	*n* (%)	
		All = 14,383.4	All = 13,377	
**Age**	0–17	203.7 (1.4)	140 (1)	0.21
	18–40	806.3 (5.6)	694 (5.2)	
	41–60	3867 (26.9)	3422 (25.6)	
	61–80	7584.3 (52.7)	7403 (55.3)	
	≥81	1922 (13.4)	1718 (12.8)	
**Gender**	Male	9394.7 (65.3)	8981 (67.1)	0.69
	Female	4988.7 (34.7)	4396 (32.9)	

**Table 2:** The percentage of urgent and elective admissions of patients with DFU or procedures associated with DFU (2017–2019 (combined and averaged data) vs. 2020).

Mode of Admission	Years	Percentage	Standard Deviation	*p*
Elective	2017–2019	29.6	0.36	0.001
	2020	26.3	0.62	
Urgent	2017–2019	67.0	0.36	0.01
	2020	73.2	0.62	

**Table 3:** A comparison of the percentage of procedures among patients with DFU in 2017–2019 (combined and averaged data) and 2020.

Type of Procedures		Years	Number/Percentage	Standard Deviation	*p*
**Amputations**	Minor	2017–2019	3146	74	0.017
		2020	4269	112.8	
	Major	2017–2019	2146	65	0.05
		2020	1600	112.6	
**Limb-salvage procedures**		2017–2019	79.4%	0.41	0.001
		2020	71.3%	0.71	

**Table 4:** Mortality among patients with DFU in 2017–2019 in comparison with 2020.

Years	Percentage	Standard Deviation	*p*
2017–2019	3.90	0.14	0.03
2020	4.84	0.25	

## Data Availability

The dataset is available on demand from the corresponding author.
